# A Novel Radar Mainlobe Anti-Jamming Method via Space-Time Coding and Blind Source Separation

**DOI:** 10.3390/s25196081

**Published:** 2025-10-02

**Authors:** Xinyu Ge, Yu Wang, Yangcheng Zheng, Guodong Jin, Daiyin Zhu

**Affiliations:** 1 Key Laboratory of Radar Imaging and Microwave Photonics, Ministry of Education, Nanjing University of Aeronautics and Astronautics, Nanjing 211116, China; gxy102@nuaa.edu.cn (X.G.); wyu@nuaa.edu.cn (Y.W.); nuaazyc@nuaa.edu.cn (Y.Z.);; 2 Shenzhen Research Institute, Nanjing University of Aeronautics and Astronautics, Shenzhen 518057, China

**Keywords:** space-time coding, blind source separation, radar mainlobe anti-jamming

## Abstract

This paper proposes a radar mainlobe anti-jamming method based on Space-Time Coding (STC) and Blind Source Separation (BSS). Addressing the performance degradation issue of traditional BSS methods under low Signal-to-Noise Ratio (SNR) and insufficient spatial resolution, this study first establishes the airborne SAR imaging geometric model and the jamming signal mixing model. Subsequently, STC technology is introduced to construct more equivalent phase centers and increase the system’s spatial Degrees of Freedom (DOF). Leveraging the increased DOFs, a JADE-based blind source separation algorithm is then employed to separate the mixed jamming signals. The separation of these signals significantly enhances the anti-jamming capability of the radar system. Simulation results demonstrate that the proposed method effectively improves BSS performance. As compared to traditional BSS schemes, this method provides an additional jamming suppression gain of approximately 10 dB in point target scenarios and about 3 dB in distributed target scenarios, significantly enhancing the radar system’s mainlobe anti-jamming capability in complex jamming environments. This method provides a new insight into radar mainlobe anti-jamming by combining the STC scheme and BSS technology.

## 1. Introduction

With the rapid development of electronic warfare technology [1], radar systems are increasingly facing complex and dynamic electromagnetic environments. Jamming signals have become more diverse and covert, posing a serious threat to radar performance. Generally, according to the propagation path of jamming signals, they can be divided into two categories: sidelobe jamming and mainlobe jamming [2]. In recent years, research on sidelobe jamming has become relatively mature. To suppress sidelobe jamming, adaptive beamforming technology based on multichannel systems [3] has been widely applied. However, the suppression of mainlobe jamming remains a considerable challenge. It is well-known that mainlobe jamming can not only lead to errors in target parameter measurement and false target tracking but also potentially cause radar system failure or even damage. Due to the spatial similarity between mainlobe jamming [4] and desired signals, existing sidelobe suppression methods struggle to achieve good suppression effects against mainlobe jamming. Therefore, how to effectively suppress mainlobe jamming has become a key issue in the field of radar anti-jamming.

Blind Source Separation (BSS) technology has, since its advent in the 1980s, fostered the development of various classic algorithms, such as Fast Independent Component Analysis (FastICA) [5], Second-Order Blind Identification (SOBI) [6], and Joint Approximate Diagonalization of Eigenmatrices (JADE). The main differences among these algorithms lie in their cost functions and optimization methods. Among them, the JADE algorithm has been widely adopted and continuously improved by researchers due to its superior separation performance and lower computational complexity. This method does not require prior knowledge of the signals or the mixing process, yet it can effectively separate the original independent source signals from multiple observed signals.

In the field of radar anti-jamming, BSS technology has also been actively applied. For instance, ref. [7] proposes an improved blind source separation algorithm based on frequency-domain robust principal component analysis (FD-RPCA-BSS) to address the problems of spatially non-stationary jamming and Signal-to-Noise Ratio (SNR) loss caused by array rotation in mainlobe jamming suppression for rotated array radar. Ref. [8] proposes a multiple mainlobe jamming suppression method based on eigen-projection processing and blind source separation, aiming to solve the problem of performance degradation in traditional BSS methods when jamming is in close proximity to the target direction. Ref. [9] proposes a mainlobe jamming suppression method based on space-time multichannel blind source separation (BSS) for array radars affected by complex mainlobe jamming. Ref. [10] proposes a mainlobe active jamming suppression method based on time-frequency domain sparse component analysis (SCA) and blind source separation (BSS), aimed at solving the problem of performance degradation in traditional methods against complex mainlobe active jamming under low-SNR conditions. This method achieves signal separation by combining Short-Time Fourier Transform (STFT) with time-frequency domain sparsity, and simulation results demonstrate its good robustness and effective suppression capability.

Despite the great potential shown by Blind Source Separation (BSS) technology [11] in mainlobe jamming suppression, its performance significantly degrades in complex jamming environments, especially in situations where the Signal-to-Noise Ratio (SNR) is low and the spatial resolution between source signals is limited. To alleviate this problem, this paper proposes a radar mainlobe anti-jamming method combining Space-Time Coding (STC) [12] with blind source separation. Space-time coding technology utilizes the spatial diversity of multiple antenna arrays to significantly increase spatial degrees of freedom and enhance the array’s spatial resolution. This characteristic effectively enhances the signal separation performance of BSS methods, including the JADE-based blind source separation algorithm, in challenging scenarios such as low SNR and insufficient spatial resolution. The JADE-based blind source separation algorithm, by constructing a fourth-order cumulant matrix and performing eigenvalue decomposition and joint diagonalization, can obtain a unitary separation matrix, thereby achieving effective separation of mixed signals [13].

The remainder of this paper is organized as follows: Section 2 details the establishment of the airborne SAR imaging geometric model and the jamming signal mixing model.

It then constructs the signal model for array-received unknown mixed signals and presents the proposed radar mainlobe anti-jamming algorithm based on blind source separation; Section 3 elaborates on the fundamental principles of space-time coding technology and analyzes its mechanism in increasing equivalent phase centers and enhancing system spatial degrees of freedom; Section 4 analyzes the simulation experimental results and compares the anti-jamming performance of the proposed method with traditional blind source separation methods in both point target and distributed target scenarios; Section 5 concludes the paper.

## 2. Theoretical Foundation

### 2.1. Signal and Jamming Model

**Figure 1:**
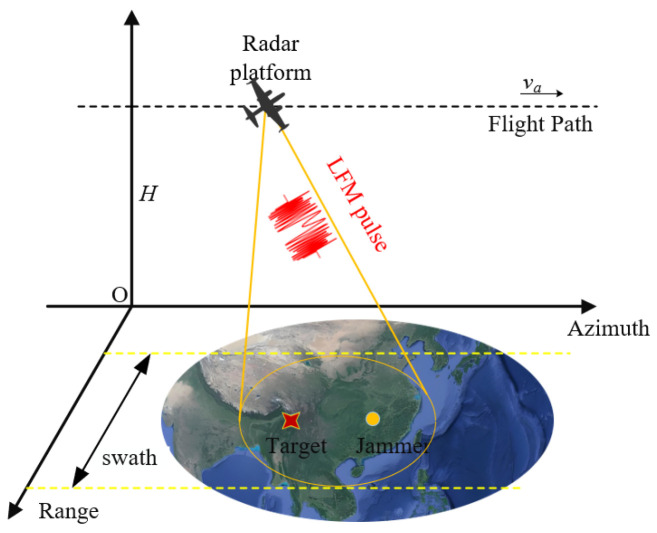
Theoretical model for airborne SAR imaging in a jamming environment.

As shown in Figure 1, the SAR platform is flying along the azimuth axis with speed $v_{a}$, the SAR platform height is H, the closet slant range is $R_{0}$, and the closest distance between the SAR platform and the target is the $t_{a}=t_{p}$ moment. We assume that the source signal is a linear frequency-modulated pulse signal:

(1) $$s(t)=\exp\left\{j2\pi \left(f_{c}t+\frac{1}{2}kt^{2}\right)\right\},$$

where T is the signal pulsewidth, $B_{r}$ is the signal bandwidth, $k=B_{r}/T$ is the frequency modulation rate, t is the range axis pulse duration, and $f_{c}$ is the central carrier frequency.

In azimuth time $t_{a}$, the baseband echo signal of real target can be expressed as follows:

(2) $$s\left(t,t_{a}\right)=\sigma_{0}\mathrm{rect}\left(\frac{t-\tau }{T}\right)\mathrm{rect}\left(\frac{t_{a}}{L/v_{a}}\right)\exp\left[j\pi k{\left(t-\tau \right)}^{2}\right]\times \exp\left(-j2\pi f_{c}\tau \right),$$

where $\sigma_{0}$ represents the scattering coefficient of a point target, L is the synthetic aperture length, $\tau =2R_{i}(t_{a})/c$ is the delay time caused by the target-to-airborne SAR distance, and c denotes the speed of light

(3) $$R_{i}\left(t_{a}\right)=\sqrt{{v_{a}}^{2}\left(t_{a}-t_{p}\right)+{R_{0}}^{2}}.$$

Deceptive jamming aims to mislead radars by transmitting false target info. Rather than just masking targets, it generates artificial echoes mimicking real ones, confusing radar ranging, velocity, or angle measurements. This may cause radars to track false targets or misjudge real ones. A key type is range deceptive jamming, which disrupts ranging via false targets at wrong distances, hindering accurate localization and identification. The expression of range deceptive jamming is as follows:

(4) $$\begin{array}{ll} J_{R}\left(t,t_{a}\right)= & \sigma_{0}\mathrm{rect}\left(\frac{t-\tau -t_{j}}{T}\right)\mathrm{rect}\left(\frac{t_{a}}{L/v_{a}}\right)\exp\left[j\pi k{\left(t-\tau -t_{j}\right)}^{2}\right]\\ & \times \exp\left[-j2\pi f_{c}\left(\tau +t_{j}\right)\right] \end{array},$$

where $t_{j}=\frac{2R_{j}}{c}$ represents the time delay of the range decoy target, $R_{j}$ is the distance from the radar to the decoy target, and c is the speed of light.

In [14], a two-dimensional controllable jamming against SAR is proposed. Two-dimensional coherent jamming can achieve controllable jamming coverage in both the range and azimuth domains, forming a regional suppression jamming effect. The signal model is given in range-frequency azimuth-time domain. It is given by the following [14]:

(5) $$\begin{array}{ll} y_{s},m(f_{r},t_{a})= & \sigma_{0}\exp\left(-j2\pi \frac{R_{i}(t_{a})}{\lambda }\right)\exp\left(-j2\pi f_{c}\Delta \tau_{s}(t_{a})\right)\\ & \times \exp\left(-j\pi \gamma_{a}{\left(t_{a}-\frac{y_{c}}{v_{a}}\right)}^{2}\right)\\ & \times \exp\left(j\pi \frac{f_{r}^{2}}{\frac{k+K_{r}^{e}}{K_{r}^{e}}k}\right)\\ & \times \exp\left(-j2\pi f_{r}\left(\frac{R_{i}(t_{a})}{c}+\Delta \tau_{s}(t_{a})\right)\right) \end{array},$$

where $R_{i}$ represent the two-way range between the reference element and the jammer, respectively. $K_{r}^{e}$ and $\gamma_{a}$ denote the chirp rate error and azimuth modulation factor, respectively, widening the mainlobe in range and azimuth to realize the jamming effect. $y_{c}$ represents the azimuth center of the blanketed area, $\Delta \tau_{s}$ represents the time delay of the suppressive jamming. $f_{r}$ represents the range frequency, and λ denotes the signal wavelength.

### 2.2. Array Signal Reception Model

To effectively perform the Blind Source Separation (BSS) algorithm, certain conditions are essential. Firstly, all source signals must be zero-mean and statistically independent. Secondly, the mixing matrix should be full column rank, and critically, the mixed signal can contain at most one Gaussian component, with all other signals being non-Gaussian.

**Figure 2:**
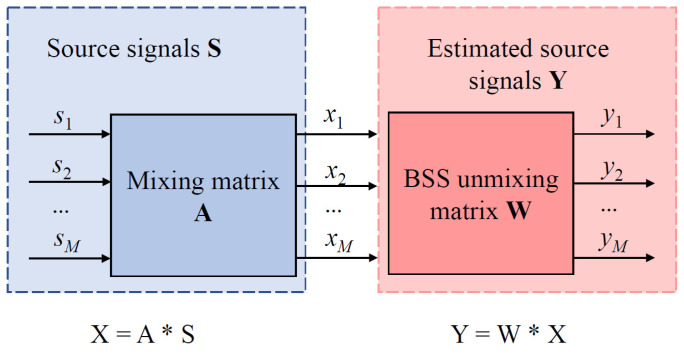
Linear transient mixed BSS model.

The linear transient hybrid model, the simplest form of the BSS algorithm, is theoretically illustrated in Figure 2.

The observed signal generated during channel transmission can be expressed as a linear transient mixture of source signals:

(6) X=AS+N,

where $S={\left\{s_{1},s_{2},\ldots ,s_{M}\right\}}^{T}$ is the unknown source signal, A is the mixing matrix, and $N={\left\{n_{1},n_{2},\ldots ,n_{M}\right\}}^{T}$ denotes Gaussian white noise introduced during channel transmission. S and N are statistically independent.

Assuming the receiving array elements are linearly arranged with a uniform inter-element spacing of d, the mixing matrix A is the equivalent receiving array manifold matrix composed of the steering vectors of the target and jamming. This matrix is an M×L full-rank matrix, i.e.,:

(7) $$A=\left(\begin{array}{cccc} 1 & 1 & \ldots & 1\\ e^{-j2\pi f_{c}d\frac{\sin\theta_{1}}{c}} & e^{-j2\pi f_{c}d\frac{\sin\theta_{2}}{c}} & \ldots & e^{-j2\pi f_{c}d\frac{\sin\theta_{L}}{c}}\\ \vdots & \vdots & \vdots & \vdots \\ e^{-j2\pi f_{c}d\frac{\left(M-1\right)\sin\theta_{1}}{c}} & e^{-j2\pi f_{c}d\frac{\left(M-1\right)\sin\theta_{2}}{c}} & \ldots & e^{-j2\pi f_{c}d\frac{\left(M-1\right)\sin\theta_{L}}{c}} \end{array}\right),$$

where in the formula, L independent source signals are incident on an M-element uniform linear array from spatial directions $\theta_{1},\theta_{2},\ldots ,\theta_{L}$ and $f_{c}$ is the frequency of the transmitted signal.

The angular resolution of an array is determined by its beamwidth, and its expression is as follows:

(8) $$\Delta \theta_{res}=k\cdot \frac{\lambda }{Nd},$$

where k is the beamforming factor, λ is the signal wavelength, d is the element channel spacing, and N is the number of subapertures.

For two targets, the resolvability requirement for their incident angles $\theta_{i}$ and $\theta_{j}$ is as follows:

(9) $$|\theta_{i}-\theta_{j}|>\theta_{res}.$$

Under conditions where the angular separation between the target and jamming sources is too close, it leads to insufficient spatial resolution. Consequently, the steering vector matrix A (formed by the target and jamming steering vectors) becomes rank-deficient, making it impossible to compute its inverse. This lack of invertibility, which is crucial for determining the separation matrix, ultimately leads to the failure of the blind source separation algorithm.

To address this issue, this paper incorporates STC into the JADE framework. The method first employs multi-transmit multi-receive (MTMR) to obtain more equivalent phase centers, thereby acquiring a greater number of virtual aperture channels. This increases the total number of received channels N, enhances angular resolution, and optimizes the separation matrix. Subsequently, the enhanced separation matrix effectively separates target signals from jamming signals.

### 2.3. Blind Source Separation Algorithms

Blind Source Separation aims to recover the original independent source signals from mixed signals without prior knowledge of the channel characteristics or specific information about the source signals. BSS algorithms based on joint matrix diagonalization, especially the classical JADE (Joint Approximate Diagonalization of Eigenmatrices) algorithm, have shown significant advantages in handling complex signal environments, particularly for mainlobe jamming suppression in radar systems.

The classical JADE blind separation algorithm [15] for received mainlobe jamming signals follows a streamlined process. First, the mixed signals are preprocessed through centering (to achieve zero mean) and whitening (for unit variance and mutual independence). Then, fourth-order cumulant matrices are computed to capture source signal independence. These cumulants are used to construct covariance matrices reflecting the source signals’ statistical characteristics. Joint diagonalization is subsequently performed to find orthogonal matrices that best diagonalize these covariance matrices. Finally, the derived separation matrix linearly transforms the mixed signals to yield the separated source signals.

**Figure 3:**
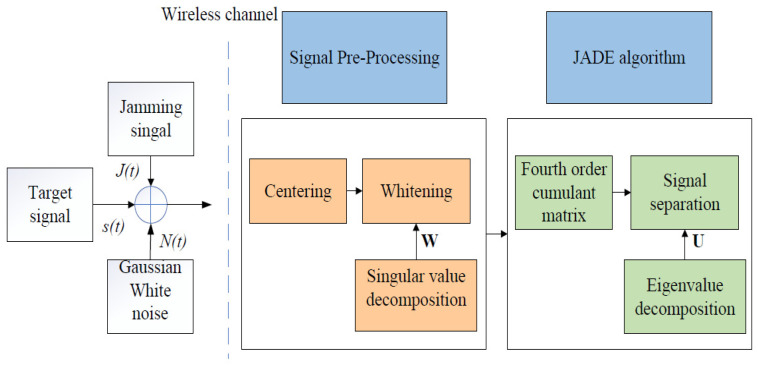
BSS flowchart based on JADE.

The principle of the JADE-BSS algorithm is illustrated in Figure 3 below:

To eliminate statistical coupling between the observed variables and enhance computational tractability, we implement signal whitening. This transformation requires a prerequisite step: centering processing. The centering operation is mathematically defined as follows:

(10) X′(t)=X(t)−E[X(t)],

where X′(t) denotes the centered observation signal satisfying E[X′(t)]=0, and E[·] represents the expectation operator over the signal ensemble or time average.

The whitening preprocessing step involves selecting a matrix W such that the covariance matrix becomes the identity matrix I. The whitening matrix W that we find using the singular value decomposition can be represent as follows:

(11) $$W=\frac{1}{\sqrt{\Lambda_{S}}}U^{H},$$

where $\Lambda_{S}$ is the singular value matrix, and U is the unitary matrix obtained from the singular value decomposition.

The purpose of whitening is twofold: it achieves data dimensionality reduction and eliminates noise while reducing the correlation between signals. Using the whitening matrix to whiten the received observation matrix X, the spatial whitened matrix is obtained as follows:

(12) Z=WX=W(AS+N)=US+WN,

where U=WA and W is the whitening matrix. To recover the source signals S, it is necessary to compute the unitary matrix U. If U is an ordinary M-dimensional matrix, the blind source separation algorithm would need to estimate $M^{2}$ parameters. However, since U is a unitary matrix, only M(M−1)/2 parameters need to be estimated. This demonstrates that whitening significantly reduces the computational complexity.

Therefore, the next step is to compute the fourth-order cumulant matrix $Q_{Z}\left(T\right)$ of the whitened signals, where the (i,j) element is defined as follows:

(13) $${\left[Q_{Z}\left(T\right)\right]}_{ij}=\sum^{p}\mathrm{cum}\left(z_{i},z_{j}^{*}z_{k},z_{l}^{*}\right){\left(T\right)}_{Ik}.$$

Here, 1<i,j<p, T is an arbitrary non-zero p×p matrix, and ${\left(T\right)}_{lk}$ denotes the element at the (l,k)−th position. The function cum(⋅) represents the operation for calculating the fourth-order cumulant.

By performing eigenvalue decomposition operation on $Q_{Z}\left(T\right)$, we can obtain the estimated V of the unitary matrix U.

(14) $$Q_{Z}\left(T\right)=V\Sigma V^{H},$$

where the Σ is the diagonal matrix of the fourth-order cumulant matrix.

Perform blind source separation on the received signals to obtain the estimated source signals:

(15) $$S=V^{H}WX.$$

From the whitening process of the JADE-BSS algorithm, it can be seen that the whitening matrix W depends on the second-order statistics of the signal X′(t):

(16) $$C_{x}=E\{X^{\prime }\left(t)X(t\right)\}=AC_{s}A^{H}+\sigma^{2}I,$$

where A is the mixing matrix, $C_{s}$ is the covariance matrix of source signals, σ represents the noise power.

Under low Signal-to-Noise Ratio (SNR) conditions, when $\sigma^{2}\gg ||AC_{s}A^{H}||$, noise will primarily influence the covariance matrix. This renders the whitened signal unable to effectively preserve target signal information. Consequently, the fourth-order cumulant matrix $Q_{Z}\left(T\right)$ becomes heavily contaminated by noise, causing the jointly diagonalized matrix Σ to lose its ideal “diagonal” property. Ultimately, this leads the optimization process to converge to an incorrect separation matrix V, resulting in separation failure.

To address this issue, a solution involves first performing matched filtering on the mixed signal to enhance the SNR, followed by the blind source separation process. Matched filtering effectively reduces noise jamming in the whitened signal, thereby optimizing the estimation of the separation matrix.

From a computational complexity perspective, the main computational overhead of the JADE-BSS algorithm includes the following aspects:

The whitening preprocessing stage requires covariance matrix calculation and singular value decomposition, with a complexity of $O(M^{2}L+M^{3})$, where M is the number of channels and L is the data length. The calculation of the fourth-order cumulant matrix, $Q_{Z}\left(T\right)$, is a core step of the algorithm, with a complexity of approximately $O(M^{4}N)$. The eigenvalue decomposition step has a complexity of $O(M^{3})$. Overall, the algorithm’s computational complexity is primarily dominated by the fourth-order cumulant calculation, at approximately $O(M^{4}N+M^{2}N)$.

Notably, while whitening increases preprocessing overhead, it effectively reduces parameter estimation complexity from $M^{2}$ to M(M−1)/2 by transforming the M×M dimensional blind source separation problem into a unitary matrix estimation. In multi-channel scenarios, the algorithm has excellent parallel processing potential. The calculation of the fourth-order cumulants can be executed in parallel by data blocks, and matrix operations like eigenvalue decomposition can also be accelerated using parallel algorithms.

## 3. Echo Separation and Implementation Strategy

**Figure 4:**
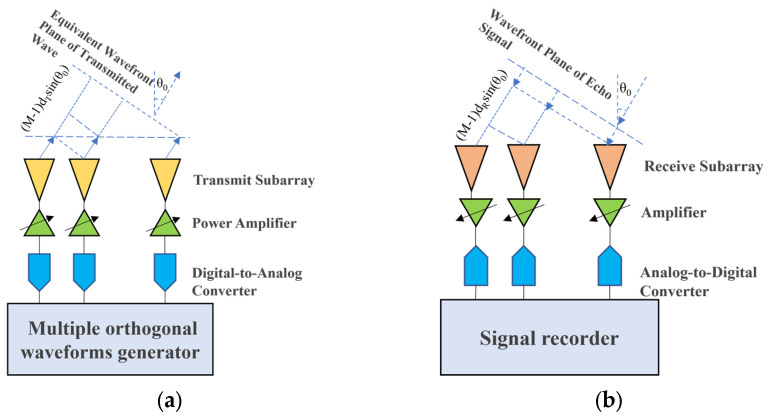
Plane wave path difference model for MIMO radar transmit and receive arrays: (**a**) transmit array with *M* channels; (**b**) receive array with *N* channels.

Compared to traditional radar systems, MIMO radar systems can provide more virtual equivalent phase centers, thereby improving system performance in aspects such as anti-jamming and HRWS (High Resolution Wide Swath) imaging. Taking the multi-channel elevation dimension as an example, as shown in Figure 4, for a centralized MIMO system with M transmitting elements and N receiving elements, each independent channel is distributed in a Uniform Linear Array (ULA) configuration along the elevation direction. For a point target with a far-field backscattering coefficient of $\sigma_{0}$ and a slant range of $r_{0}$ (distance from the center of the reference channel), and an angle of arrival of $\theta_{0}$, the waveform received by the $n^{th}$ channel can be expressed as follows:

(17) $$\begin{array}{ll} S_{n}= & \sigma_{0}\sum_{m=1}^{M}\mathrm{rect}\left(t-\frac{r_{n}+r_{m}}{c}\right)\times s_{m}\left(t-\frac{r_{n}+r_{m}}{c}\right)\\ & \times \exp\left(2j\pi f_{c}\left(t-\frac{r_{n}+r_{m}}{c}\right)\right) \end{array},$$

where $r_{n},r_{m}$ represents the distance from each channel to the point target, $s_{m}$ is the transmitted waveform of the $m^{th}$ channel, and $f_{c}$ is the carrier frequency. For orthogonal waveforms, the echo signals from each transmit channel can be separated directly by waveform cross-correlation, and for fast time t, $\frac{r_{n}+r_{m}}{c}\approx \frac{2r_{0}}{c}$, after down-conversion and cross-correlation, we can obtain the following:

(18) $$S_{m,n}=\sigma_{0}\mathrm{rect}\left(t-\frac{2r_{0}}{c}\right)\times p_{m}\left(t-\frac{2r_{0}}{c}\right)\times \exp\left(2j\pi \frac{r_{n}+r_{m}}{\lambda }\right),$$

where $P_{m}$ represents the autocorrelation result of waveform m, and λ is the wavelength. Furthermore, under the far-field assumption, according to the plane wave theory model, the above equation can be further decomposed into:

(19) $$\begin{array}{ll} S_{m,n}\approx & \sigma_{0}\mathrm{rect}\left(t-\frac{2r_{0}}{c}\right)\times p_{m}\left(t-\frac{2r_{0}}{c}\right)\\ & \times \exp\left(2j\pi \frac{d_{m}\sin\theta_{0}+d_{n}\sin\theta_{0}}{\lambda }\right) \end{array},$$

where $\left\{\begin{array}{l} d_{n}=(n-1)d_{R}\\ d_{m}=(m-1)d_{R} \end{array}\right.$, $d_{R}$ is the receiving channel spacing; we thus have the following:

(20) $$\begin{array}{ll} S_{m,n} & \approx \sigma_{0}\mathrm{rect}\left(t-\frac{2r_{0}}{c}\right)\times p_{m}\left(t-\frac{2r_{0}}{c}\right)\times \exp\left(2j\pi \frac{\left(\left(m-1\right)+\left(n-1\right)\right)d_{R}\sin\theta_{0}}{\lambda }\right)\\ & =\sigma_{0}\mathrm{rect}\left(t-\frac{2r_{0}}{c}\right)\times p_{m}\left(t-\frac{2r_{0}}{c}\right)\times \exp\left(2j\pi \frac{2\left(\frac{m+n}{2}-1\right)d_{R}\sin\theta_{0}}{\lambda }\right) \end{array}.$$

That is, the echo signal for an $m^{th}$ channel transmit and $n^{th}$ channel receive is equivalent to a single-transmit single-receive result from a virtual channel located at the physical center of the two channels. From this, we obtain the following array configuration: a traditional Uniform Planar Array (UPA) uses a 4 × 4 physical antenna layout (Configuration 1), achieving symmetric beamforming for elevation and azimuth angles through regular arrangement. To further increase the number of channels in the system, this paper introduces Multiple-Input Multiple-Output (MIMO) technology [16]. By deploying multiple transmit and receive antennas, MIMO can generate more equivalent phase centers, thereby virtually expanding the array aperture. After waveform separation [17], the echo received by the $n^{th}$ receive channel from the signal transmitted by the $m^{th}$ transmit channel can be represented as follows:

(21) $$x_{m,n}(\tau )=\gamma_{0}\delta \left(\tau -\frac{2r_{0}}{c}\right)e^{-j2\pi f_{0}\frac{2r_{0}}{c}}e^{j2\pi f_{0}\frac{(m-1)d_{T}+(n-1)d_{R}}{c}\sin\left(\theta_{0}\right)},$$

where $\gamma_{0}\in C$, $r_{0}$, $\theta_{0}$, c, $f_{0}$, $d_{T}$, and $d_{R}$ denote the back scattered-coefficient of point-like target, the slant-range, the incident-angle, the speed of light, the carrier frequency, the transmitting channel spacing, and the receiving channel spacing respectively.

Without loss of generality, for a 4-transmit and 16-receive MIMO architecture, two virtual arrays can be constructed: (1) the transmitting antennas are arranged along the elevation direction, the equivalent phase centers are extended to 7 channels in the azimuth dimension, forming a 4 (elevation) × 7 (azimuth) configuration (termed as Configuration 2); (2) the transmitting antennas are distributed along the azimuth direction, the number of equivalent elevation channels increases to 7, resulting in a 7 (elevation) × 4 (azimuth) configuration (termed as Configuration 3). As compared to the current single-input multiple-output (SIMO) system, the MIMO architecture can provide more spatial degrees of freedom.

**Figure 5:**
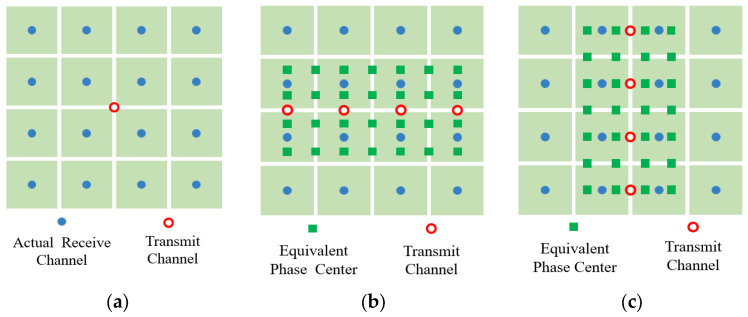
Array configuration diagram: (**a**) Configuration 1; (**b**) Configuration 2; (**c**) Configuration 3.

With the employment of MIMO technique [18,19,20], we can obtain the following array configurations, as shown in Figure 5:

## 4. Experimental Results and Analysis

**Figure 6:**
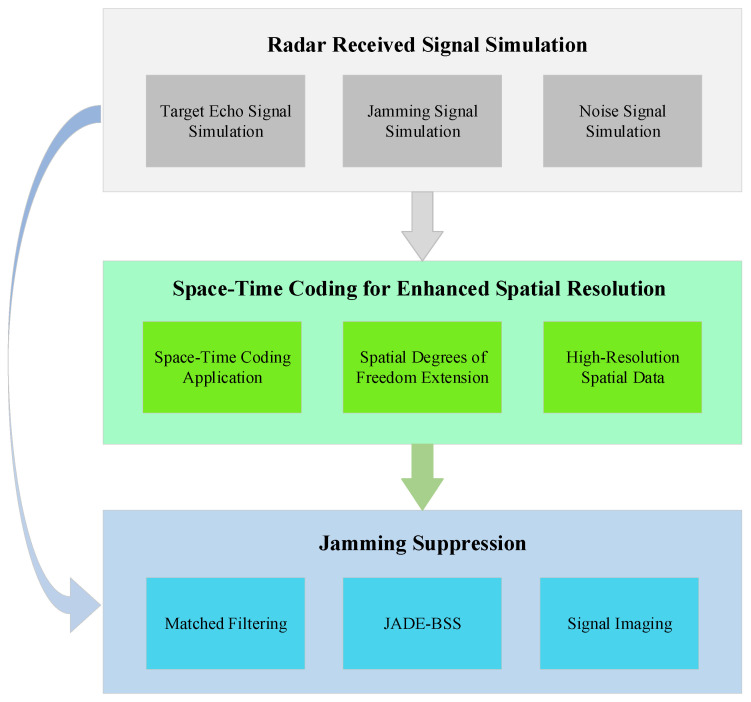
Flowchart of anti-suppression jamming operation.

To evaluate the proposed method’s effectiveness, we conducted simulation experiments under two scenarios: point targets and distributed targets. Mixed radar signals were simulated using MATLAB (version 2022b), then processed through both the proposed approach and the conventional JADE-BSS algorithm for blind source separation. The separated signals underwent Range-Doppler Algorithm (RDA) processing for image reconstruction. Performance comparison using the Jamming Suppression Ratio (JSR) as the evaluation metric demonstrated the relative anti-jamming capabilities of each method. The complete experimental workflow is depicted in Figure 6.

To evaluate the performance of our proposed anti-jamming method, we employ the JSR as the evaluation metric. JSR quantifies the effectiveness of jamming suppression by calculating the ratio of the input power to the output power of the jamming signal. Its calculation formula is as follows:

(22) $$\mathrm{JSR}(\mathrm{dB})=10\cdot \log_{10}\left(\frac{P_{i,out}}{P_{i,in}}\right),$$

where $P_{i,in}$ and $P_{i,out}$ represent the power of jamming before and after the suppression process, respectively. A larger Jamming Suppression Ratio value indicates better jamming suppression performance. Table 1 show the simulation parameters.

### 4.1. Point-like Target Simulation

To validate the effectiveness of the proposed STC-JADE blind source separation algorithm, simulation experiments were designed comparing three distinct array configurations. Configuration 1 employed a conventional single-transmit, 16-receive (1T16R) array with 4 channels in both elevation and azimuth dimensions, serving as a performance baseline using the traditional JADE algorithm. Conversely, Configurations 2 and 3 both utilized an equivalent four-transmit, 16-receive (4T16R) array, leveraging space-time coding (STC) to achieve enhanced spatial degrees of freedom in the azimuth (4 × 7 channels) and elevation (7 × 4 channels) dimensions, respectively, with the proposed STC-JADE algorithm applied. This multi-transmit, multi-receive space-time coding scheme significantly increases the number of virtual array elements from 16 to 28, thereby substantially improving the array’s spatial resolution compared to conventional single-transmit, multi-receive systems. The experiments quantitatively assessed the blind source separation performance of these configurations by calculating the JSR, thereby validating the STC-JADE algorithm’s performance advantage over the traditional JADE algorithm.

Assume the radar receives a target signal and a range deception jamming signal, with the jammer located near the target. The target direction is [26.5°, 43°] (elevation, azimuth), and the jammer direction is [28°, 41.5°]. The spatial coordinates of the radar, target, and jammer are listed in Table 2 (units: meters).

The frequency-modulated noise jamming signal is expressed as follows:

(23) $$J(t)=U_{j}\exp\left[j\left(2\pi f_{c}t+2\pi k\int_{0}^{t}u(t)dt+\phi \right)\right],$$

where $U_{j}$ is carrier frequency amplitude, k is the frequency modulation slope, u(t) is the Gaussian white noise with zero mean whose variance is $\sigma^{2}$, and ϕ is the phase of random distribution.

**Figure 7:**
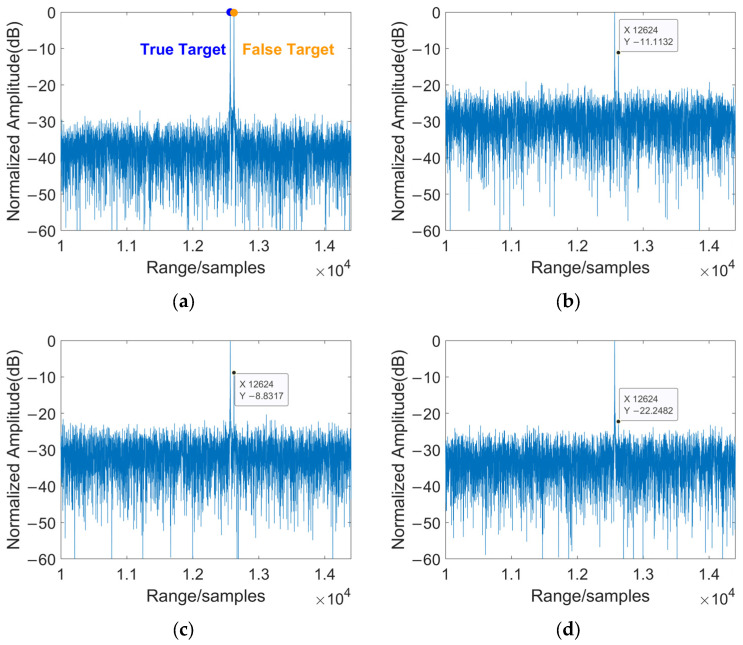
Separation results before and after typical suppressive jamming for measured data: (**a**) results of received mixed signals; (**b**) separation results of JADE method; (**c**) separation results of STC-JADE (7 × 4) method; (**d**) separation results of STC-JADE (4 × 7) method.

Figure 7 illustrates the separation results before and after jamming suppression. As shown, the original signal contains both the target and jamming signals, with the latter making the target signal difficult to clearly identify. Observing each subplot in Figure 7 reveals that blind source separation based on traditional array configurations (i.e., Configuration 1) yields limited effectiveness, with noticeable jamming signals still present. However, Configuration 2, which leverages space-time coding to achieve more degrees of freedom, shows only a single prominent peak in its blind source separation result. This indicates that the target signal was successfully isolated after the jamming suppression processing.

**Figure 8:**
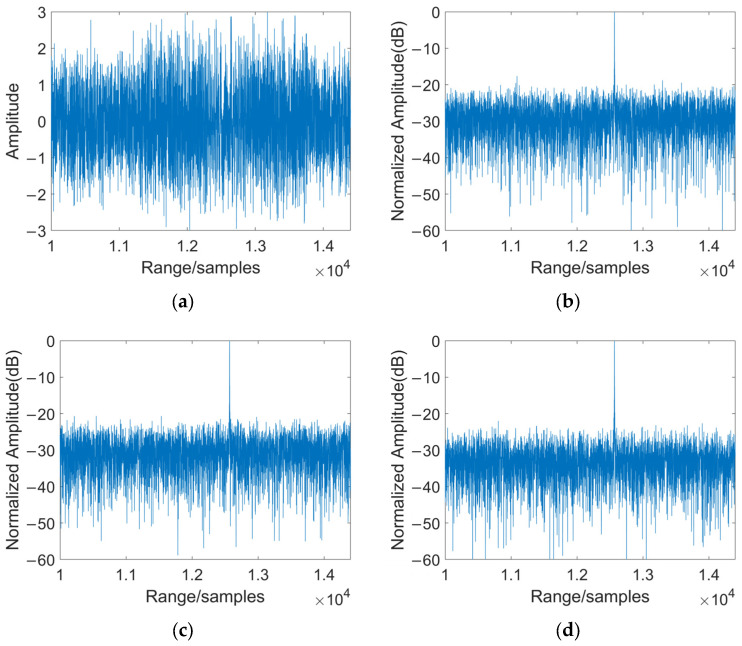
Separation results before and after anti-NFM jamming for measured data: (**a**) results of received mixed signals; (**b**) separation results of JADE method; (**c**) separation results of STC-JADE (7 × 4) method; (**d**) separation results of STC-JADE (4 × 7) method.

Figure 8 shows the separation results before and after typical barrage jamming suppression. As shown in Figure 8a, the target echo signal is completely submerged in jamming noise, making it impossible to identify the target. Figure 8b–d display the processing results of different jamming suppression methods. It can be seen that after processing, the signal quality is significantly improved and the jamming is effectively suppressed.

Simulation experiments demonstrate that the STC-JADE method significantly enhances blind source separation performance compared to the traditional JADE algorithm. Space-time coding effectively improves the array’s spatial resolution by providing additional degrees of freedom and generating extra equivalent phase centers, resulting in at least a 10 dB improvement in the JSR. This optimized selection not only maximizes the advantages of MIMO technology but also further strengthens blind source separation performance and the system’s anti-jamming capability.

### 4.2. Distributed Scene Simulation

**Figure 9:**
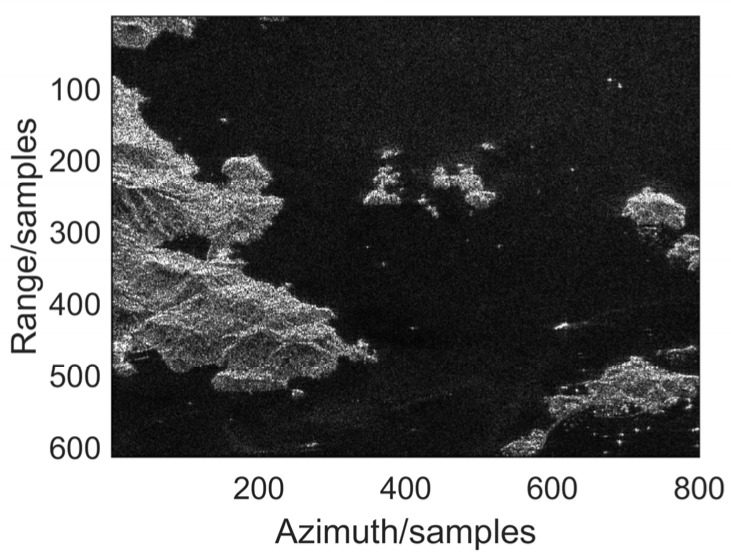
Origin distributed scene simulation.

The radar platform parameters in this section are the same as those used in the dot target simulation. To further verify the practicality of the proposed method, this subsection conducted distributed scene simulation experiments. The echo is generated utilizing a SAR image. We added two-dimensional coherent jamming to the echo data to simulate imaging data where jamming needs to be suppressed. Figure 9 shows the image results of the original simulated scene. The highlighted parts in the image are ships, the black parts are the sea, and the gray parts are islands.

**Figure 10:**
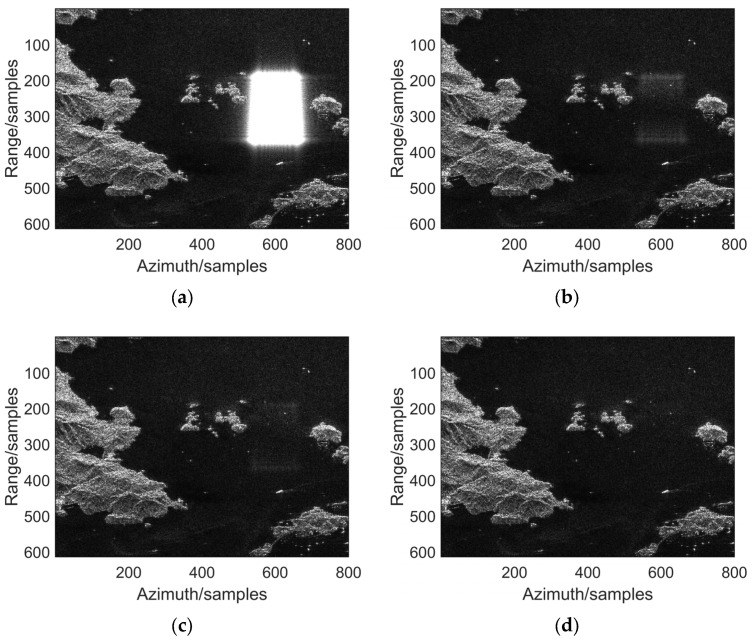
Imaging results before and after anti-two-dimensional coherent jamming: (**a**) image before anti-jamming; (**b**) anti-jamming results of JADE method; (**c**) anti-jamming results of STC-JADE (4 × 7) method; (**d**) anti-jamming results of STC-JADE (7 × 4) method.

The anti-jamming performance of the traditional JADE method and our proposed STC-JADE method on echo data was investigated. Figure 10a presents the SAR imaging results under two-dimensional coherent suppression jamming. Compared to Figure 9, it can be observed that the suppression jamming forms a distinct strong-amplitude rectangular occlusion area at the center of the imaging region, which completely masks the underlying target information and severely degrades the imaging quality. Figure 10 shows the signal waveforms before and after two-dimensional coherent suppression jamming.

From Figure 10, it can be observed that after applying the traditional JADE anti-jamming method, most jamming signals are effectively suppressed, and the scattering characteristics of the target area are partially restored. Compared to the suppression jamming results, target information reappears in the previously completely obscured central region, although slight residual jamming effects are still present. After utilizing the proposed STC-JADE anti-jamming method, the imaging results show high similarity to the ideal image under jamming-free conditions, demonstrating superior anti-jamming performance. From Figure 10d, it can be seen that our proposed method exhibits high signal reproducibility and low signal loss.

**Figure 11:**
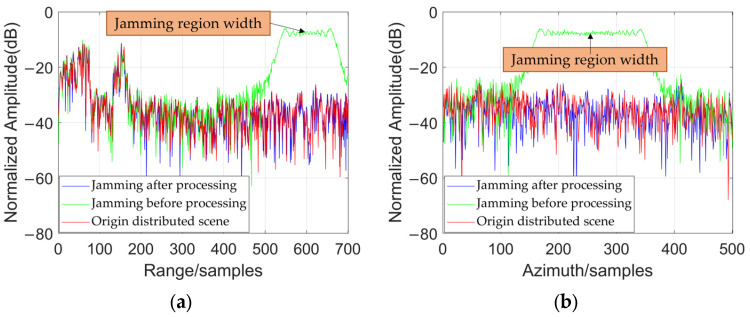
The profiles of jamming before and after process: (**a**) profiles along range; (**b**) profiles along azimuth.

To quantitatively evaluate the jamming suppression effectiveness, Figure 11 presents a comparison of the jamming profiles along the range and azimuth directions before and after applying the proposed method, showing a significant reduction in the separated jamming components. The JSR of this method for suppressing jamming is 23.1 dB. This indicates a substantial improvement in the system’s anti-jamming performance.

Table 3 provides the Jamming Suppression Ratios for different methods under equivalent conditions. Compared to the traditional JADE method, the proposed STC-JADE method achieves a jamming suppression improvement of approximately 3.6 dB.

Experimental results clearly demonstrate the significant advantages of our proposed STC-JADE method over the traditional JADE method. This is primarily due to Space-Time Coding (STC) technology, which enhances spatial resolution by effectively utilizing the array’s spatial degrees of freedom, thereby substantially improving blind source separation performance.

The study further reveals that the algorithm’s performance is closely tied to the array’s geometric configuration. Different array row and column layouts directly affect their resolution in spatial angles. This difference in resolution determines the blind source separation algorithm’s ability to effectively distinguish between desired signals and interference, ultimately impacting the final separation outcome.

## 5. Conclusions

This study addresses the performance degradation of the traditional Joint Approximate Diagonalization of Eigenmatrices algorithm for radar mainlobe anti-jamming under conditions of low spatial resolution and low signal-to-noise ratio. To overcome this limitation, we propose a STC based JADE blind source separation method to counter mainlobe jamming. By introducing STC technology, this method effectively expands the system’s spatial degrees of freedom, thereby enhancing spatial resolution and significantly improving blind source separation performance. Detailed simulation experiments were conducted to verify the effectiveness of the proposed method. The results demonstrate that, compared to traditional BSS schemes, our STC-JADE method achieves an improved jamming suppression gain of approximately 10 dB in point target scenarios and a performance improvement of about 3.6 dB in distributed target scenarios. This substantially enhances the radar system’s mainlobe anti-jamming capability in complex electromagnetic environments. Specifically, while the traditional JADE method suppresses jamming, it still leaves noticeable residual effects, leading to suboptimal target imaging quality; in contrast, the imaging results from our proposed method show high similarity to the ideal image under jam-free conditions, fully demonstrating its superior anti-jamming performance and effective recovery of target information. This research provides a practical technical solution for radar systems to counter mainlobe jamming.

## Figures and Tables

**Table 1:** Radar parameter configuration.

Parameters	Values
Reference carrier frequency	10 GHz
Pulse bandwidth	100 MHz
Pulse width	10 µs
Sampling rate	120 MHz
PRF	600 Hz
Platform height	3 km
Platform Velocity	150 m/s
Scene center slant range	10 km
SNR	6 dB

**Table 2:** Coordinate parameters of target, jammer, and false target.

Target	Jammer	False Target
(1020,1100,0)	(1056,1200,0)	(1220,1000,0)

**Table 3:** JSR for different methods under equivalent conditions.

Array Configuration	Config. 1 (Traditional JADE)	Config. 2 (STC-JADE, 4 × 7)	Config. 2 (STC-JADE, 7 × 4)
JSR (dB)	19.5	20.6	23.1

## Data Availability

The original contributions presented in this study are included in the article. Further inquiries can be directed to the corresponding author.
